# Supplementary material for: Changes in physicochemical properties and microbial community succession during leaf stacking fermentation
Source: AMB Express. 2023 Nov 22;13:132. doi: 10.1186/s13568-023-01642-8 (PMC10665287; doi:10.1186/s13568-023-01642-8)

Supplementary Material

Journal Name: AMB Express

Article Title: Changes in physicochemical properties and microbial community succession during leaf stacking fermentation

Authors: Guanghai Zhang, Lu Zhao, Wei Li, Heng Yao, Canhua Lu, Gaokun Zhao, Yuping Wu, Yongping Li, Guanghui Kong*

*** Correspondence:**

Guanghui Kong, 13908776036@163.com, Yunnan Academy of Tobacco Agricultural Sciences, Kunming, Yunnan, 650021, China

Fig. S1 The rarefaction curves of 54 samples bacteria (A) and fungi (B).

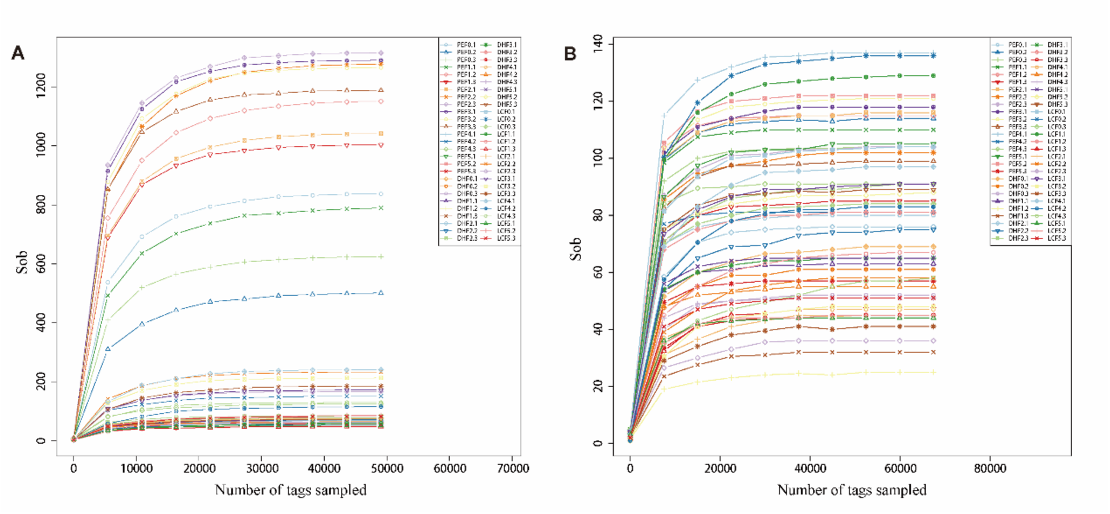

Fig. S2 This Venn diagram shows specific OTUs and shared OTUs of bacteria (A) and fungi (B) among different origins.

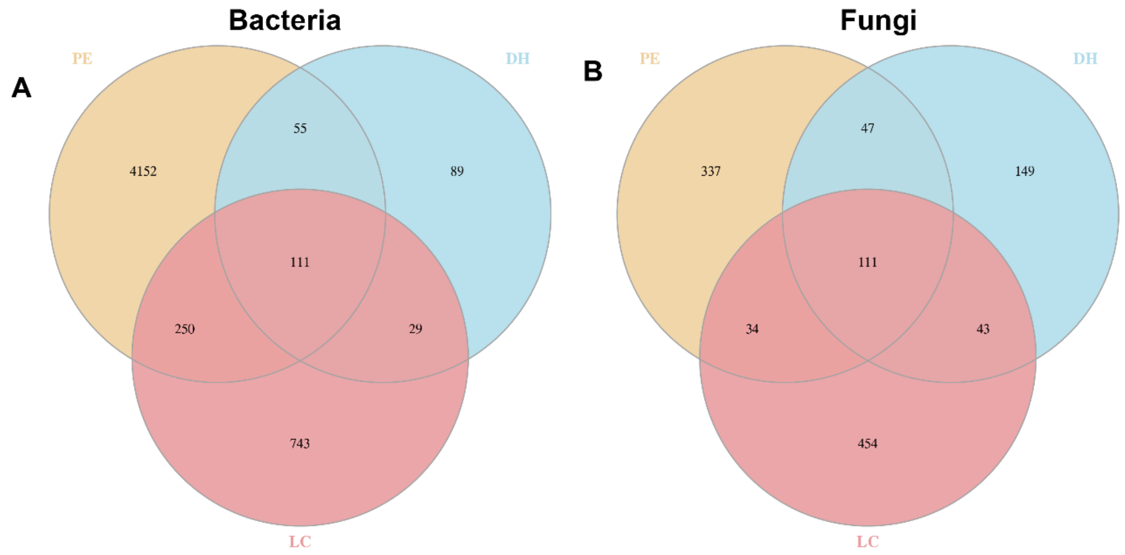

Fig. S3 The core taxa can distinguish fermentation stages of CTLs. Classification of random forest models of the fermentation stage of the core taxa of LC bacteria (A) and fungi (B). Shown are the important features (top 20) based on Mean Decrease Gini (MDG) of random forest models of the core taxa of LC bacteria (C) and fungi (D).

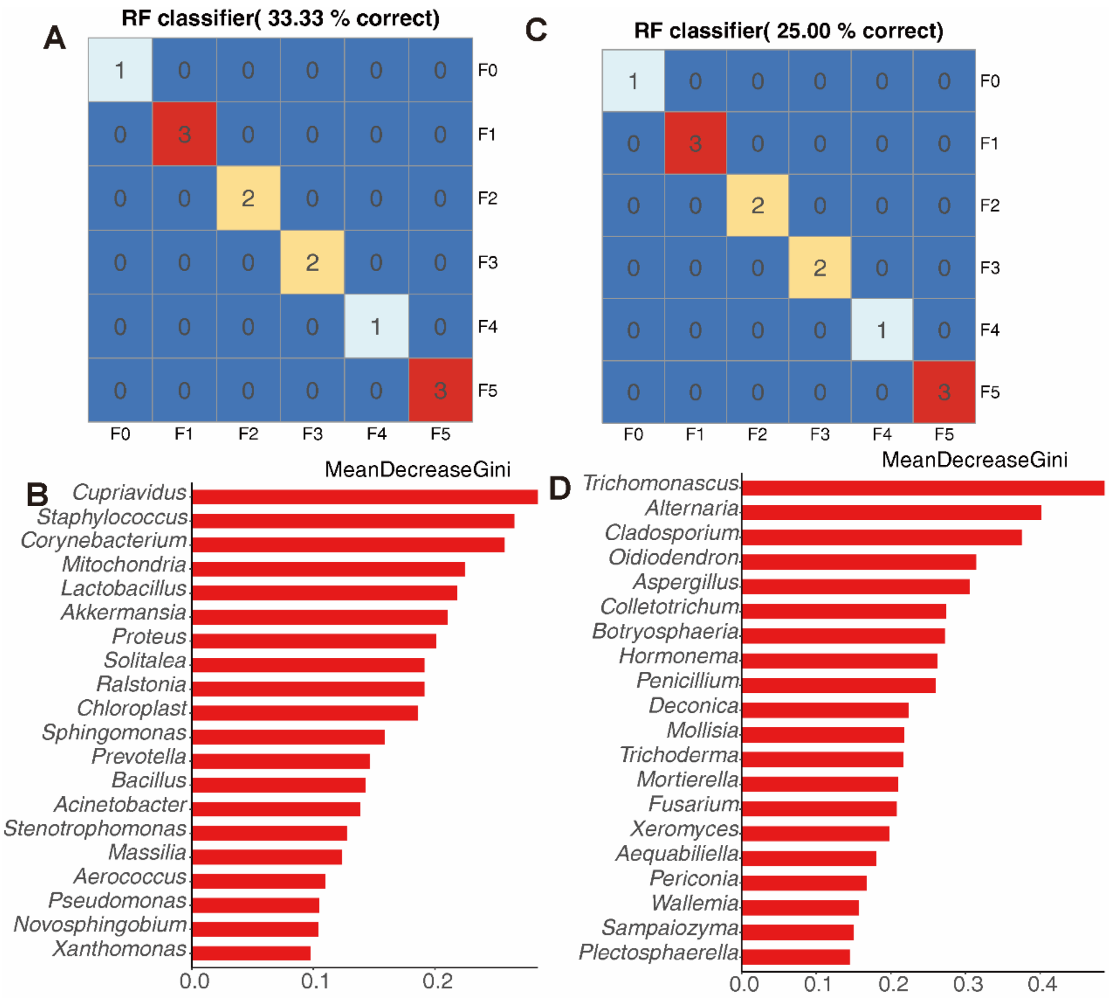

Figure. S4 The core taxa can distinguish fermentation stages of CTLs. Classification of random forest models of the fermentation stage of the core taxa of DH bacteria (A) and fungi (B). Shown are the important features (top 20) based on Mean Decrease Gini (MDG) of random forest models of the core taxa of DH bacteria (C) and fungi (D).

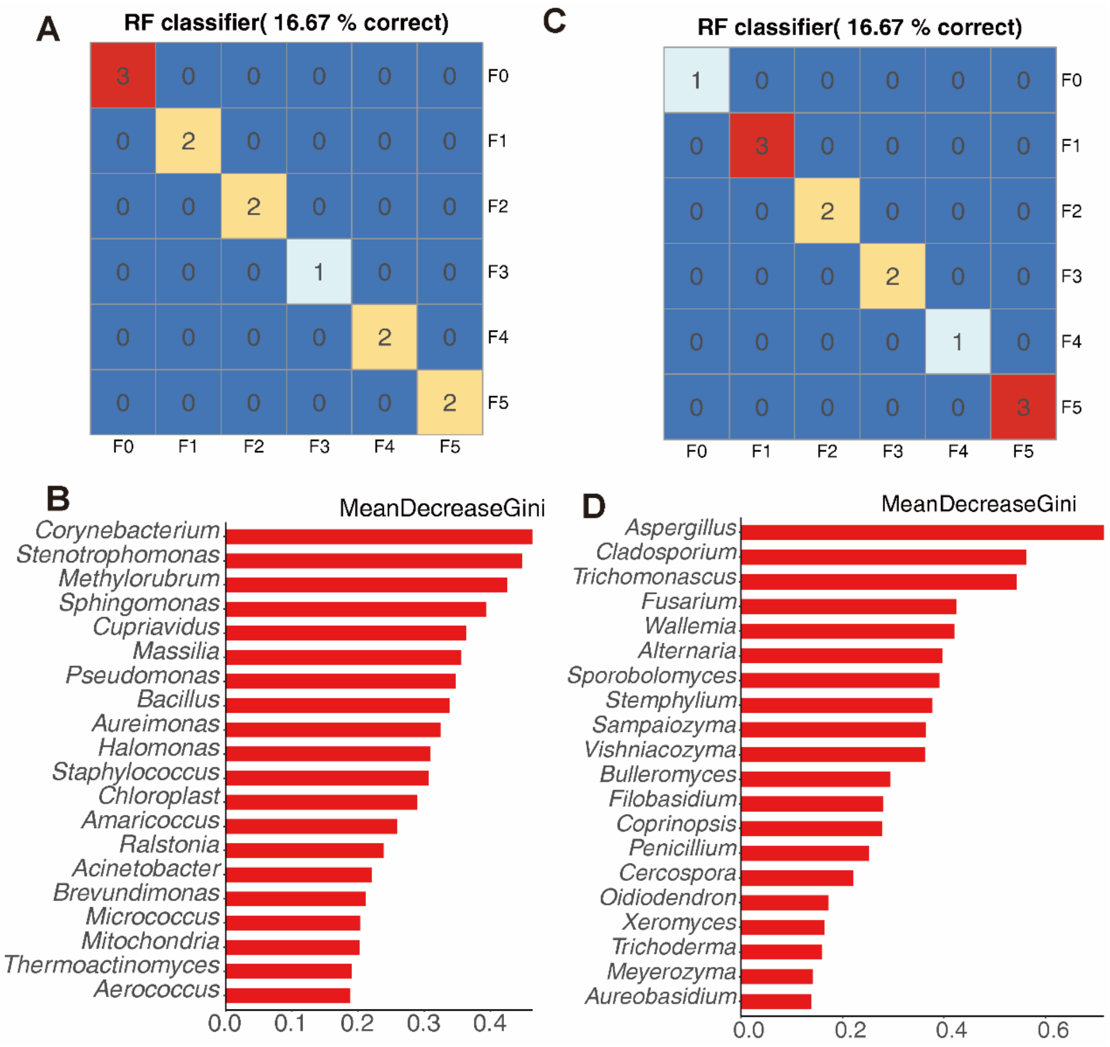

Fig. S5 CCA reflected the relationship between metabolic enzymes (A, bacteria; D, fungi), physicochemical properties (B, bacteria; E, fungi), volatile aroma compounds (C, bacteria; F, fungi) and microbial community composition during cigar tobacco leaves fermentation.

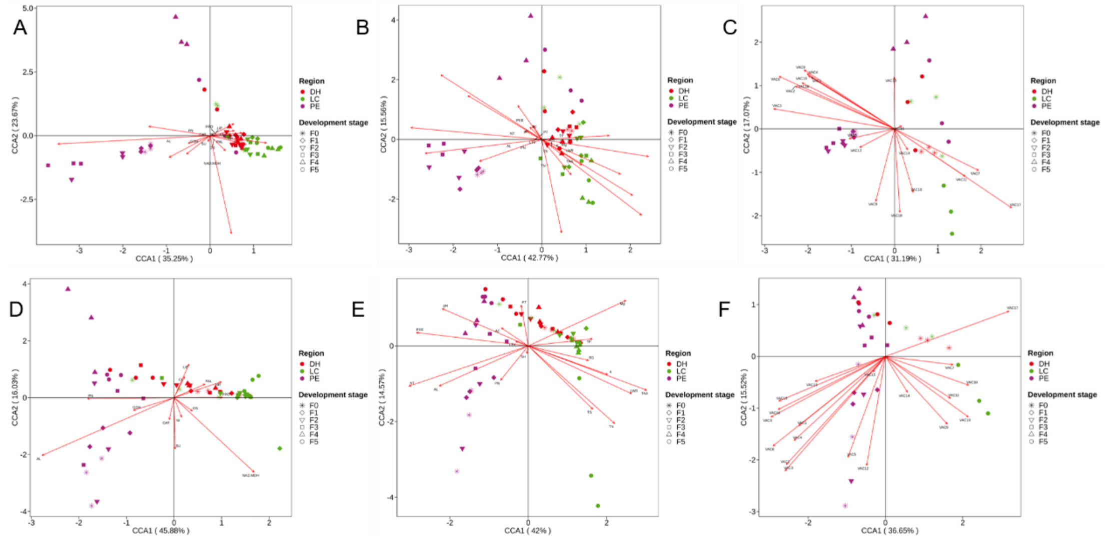

Fig. S6 Network analysis based on the co-occurrence of physicochemical properties (A, B)，metabolic enzymes(C，D) and the bacterial and fungal communities. Purple vee nodes represent volatile components. Grey ellipse nodes represent microbial members. Direct connections between nodes indicate strong correlations (Pearson correlation coefficient, P < 0.05). Red lines represent positive interactions between nodes, and blue lines represent negative interactions. The sizes of vee nodes represent the interconnected degree. The sizes of circle nodes represent the average relative abundances of bacteria and fungi.

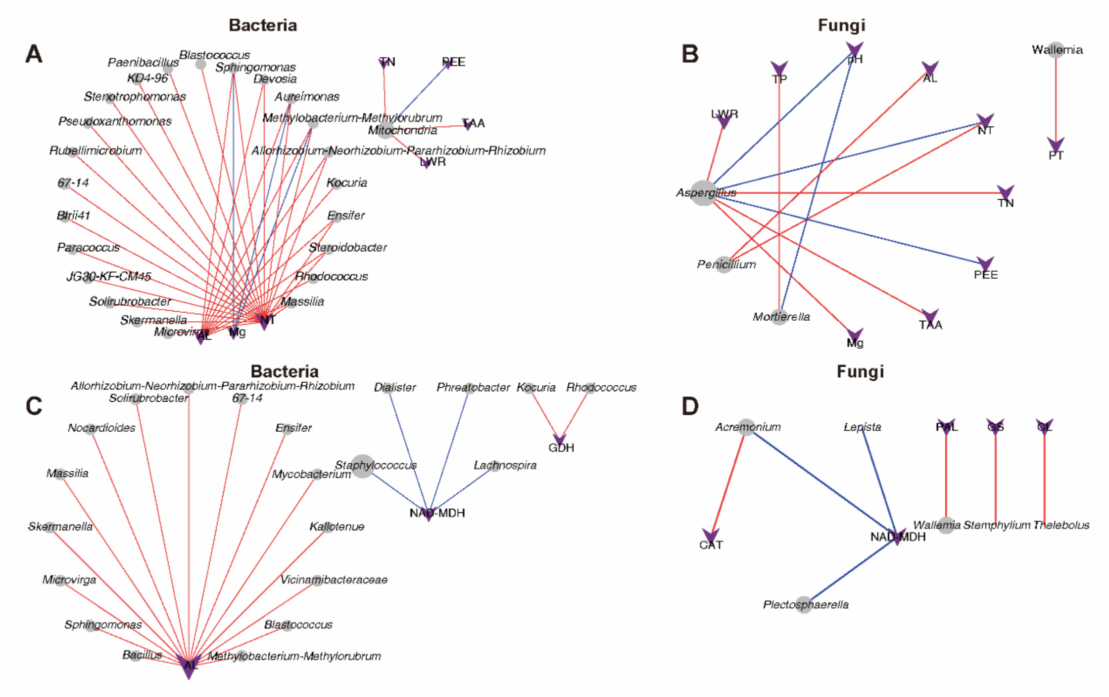

Supplement: Supplementary file 1 — Supplementary Material 1 [file 13568_2023_1642_MOESM1_ESM.pdf]
